# Proarrhythmic Electrical Remodeling by Noncardiomyocytes at Interfaces With Cardiomyocytes Under Oxidative Stress

**DOI:** 10.3389/fphys.2020.622613

**Published:** 2021-02-02

**Authors:** Yali Zhao, Shankar Iyer, Maryam Tavanaei, Nicole T. Nguyen, Andrew Lin, Thao P. Nguyen

**Affiliations:** Division of Cardiology, Department of Medicine, The Cardiovascular Research Laboratory, David Geffen School of Medicine at UCLA, Los Angeles, CA, United States

**Keywords:** fibrosis, remodeling, oxidative stress, H_2_O_2_, cardiomyocyte, myofibroblast, interface, arrhythmia

## Abstract

Life-threatening ventricular arrhythmias, typically arising from interfaces between fibrosis and surviving cardiomyocytes, are feared sequelae of structurally remodeled hearts under oxidative stress. Incomplete understanding of the proarrhythmic electrical remodeling by fibrosis limits the development of novel antiarrhythmic strategies. To define the mechanistic determinants of the proarrhythmia in electrical crosstalk between cardiomyocytes and noncardiomyocytes, we developed a novel *in vitro* model of interface between neonatal rat ventricular cardiomyocytes (NRVMs) and controls [NRVMs or connexin43 (Cx43)-deficient HeLa cells] vs. Cx43^+^ noncardiomyocytes [aged rat ventricular myofibroblasts (ARVFs) or HeLaCx43 cells]. We performed high-speed voltage-sensitive optical imaging at baseline and following acute H_2_O_2_ exposure. In NRVM-NRVM and NRVM-HeLa controls, no arrhythmias occurred under either experimental condition. In the NRVM-ARVF and NRVM-HeLaCx43 groups, Cx43^+^ noncardiomyocytes enabled passive decremental propagation of electrical impulses and impaired NRVM activation and repolarization, thereby slowing conduction and prolonging action potential duration. Following H_2_O_2_ exposure, arrhythmia triggers, automaticity, and non-reentrant and reentrant arrhythmias emerged. This study reveals that myofibroblasts (which generate cardiac fibrosis) and other noncardiomyocytes can induce not only structural remodeling but also electrical remodeling and that electrical remodeling by noncardiomyocytes can be particularly arrhythmogenic in the presence of an oxidative burst. Synergistic electrical remodeling between H_2_O_2_ and noncardiomyocytes may account for the clinical arrhythmogenicity of myofibroblasts at fibrotic interfaces with cardiomyocytes in ischemic/non-ischemic cardiomyopathies. Understanding the enhanced arrhythmogenicity of synergistic electrical remodeling by H_2_O_2_ and noncardiomyocytes may guide novel safe-by-design antiarrhythmic strategies for next-generation iatrogenic interfaces between surviving native cardiomyocytes and exogenous stem cells or engineered tissues in cardiac regenerative therapies.

## Introduction

In the normal adult mammalian left ventricle, electrically excitable (capable of firing all-or-none action potentials) cardiomyocytes comprise 80% of the volume but only 30% of the cell number (Vliegen et al., 1991). In contrast, ventricular noncardiomyocytes (predominantly fibroblasts and endothelial cells) are electrically non-excitable (incapable of firing action potentials), but they outnumber cardiomyocytes and comprise the cellular majority in the heart. Following fibrotic structural remodeling, the ratio of noncardiomyocytes to cardiomyocytes increases further, and the noncardiomyocyte composition changes as new active myofibroblasts over-replace old quiescent fibroblasts. Myofibroblasts are noncardiomyocytes that share features with both cardiomyocytes and fibroblasts. Myofibroblasts do not exist in healthy young myocardium. They only proliferate in the injured, diseased, aged, or therapeutically ablated myocardium, by phenotypic conversion of existing fibroblasts, epithelial–mesenchymal transition, or *de novo* production. Fibrosis develops as myofibroblasts deposit collagen bundles to salvage the compromised cardiac mechanical function, albeit at the cost of higher risk of life-threatening ventricular arrhythmias (Nguyen et al., 2014).

Traditionally, the role of myofibroblasts in arrhythmogenesis was deemed purely indirect and passive, attributed to the interference of collagen bundles with electrical propagation and source–sink relationships (Kucera et al., 1998). However, the last 15 years marks a growing recognition for a more direct and active role of myofibroblasts in arrhythmogenesis. Myofibroblasts may directly engage local cardiomyocytes in bidirectional crosstalk by secreting soluble paracrine factors (Pedrotty et al., 2009; Vasquez et al., 2010; Cartledge et al., 2015). They may also form connexin (Cx)-based gap junction coupling for short-range heterotypic intercellular connectivity or Cx-based nanotube coupling for long-range heterotypic intercellular connectivity (Gaudesius et al., 2003; He et al., 2011; Nguyen et al., 2012; Mahoney et al., 2016; Quinn et al., 2016; Ribeiro-Rodrigues et al., 2017; Rubart et al., 2018). Additionally, these two mechanisms may synergize. For example, soluble myofibroblast paracrine factors, such as TGF-β1, may promote heterotypic gap junction coupling and increase myofibroblast arrhythmogenicity (Salvarani et al., 2017). Yet how fibrosis causes proarrhythmic electrical remodeling of local cardiomyocytes is not completely understood.

We hypothesized that myofibroblasts are not unique in proarrhythmic remodeling of cardiomyocyte electrophysiology via electrical crosstalk, as other non-excitable cells may also be capable if they are equipped to conduct adequate intercellular electrical communication and are more depolarized than cardiomyocytes. To test this hypothesis, we selected three noncardiomyocyte cell types based on extensive literature evidence of the differences in their Cx43 expression, which is negligible in HeLa cells, moderate in myofibroblasts (higher than in fibroblasts), and high in HeLa cells engineered to overexpress Cx43 (HeLaCx43). Literature is replete with immunohistochemistry and cutting-edge imaging evidence of *in vitro* Cx43 detection at cardiomyocyte short- and long-range interfaces with fibroblasts and HeLaCx43 cells (Gaudesius et al., 2003; Miragoli et al., 2006; He et al., 2011; Quinn et al., 2016; Ribeiro-Rodrigues et al., 2017). However, immunohistochemical evidence of Cx presence (or absence) cannot prove (nor disprove) that functional gap junctions/nanotubes formed or that their function remained unchanged for the experimental duration. Indeed, like other ion channels, both the formation and functional state (open vs. closed) of gap junctions/nanotubes are dynamic, clinically and experimentally. They are transient by nature (Palacios-Prado and Bukauskas, 2009) and depend on a host of variables, including local environmental stress, pH, and local tissue stretch, just to name a few. Unfortunately, evidence of heterotypic gap junction function, and more importantly, of potential attendant functional consequences and clinical relevance is scant. Therefore, the objective of this study is to address this knowledge gap in the field. Furthermore, we postulated that local environmental stress, such as an acute H_2_O_2_-mediated oxidative burst, might synergize by further lowering the repolarization reserve of cardiomyocytes and the protective source-sink mismatch of the myocardium. This study aimed to provide functional proof-of-concept for our hypotheses using a novel *in vitro* model of cardiomyocyte–noncardiomyocyte interface that we developed.

## Methods

The experimental protocol was approved by the University of California, Los Angeles (UCLA) Institutional Animal Care and Use Committee and conformed to the National Institutes of Health (NIH) *Guide for the Care and Use of Laboratory Animals*.

### Isolating Aged Rat Ventricular Myofibroblasts

Ventricular myofibroblasts were freshly isolated from aged (24- to 26-month-old) Fischer 344 (F344) rats (ARVFs) of the National Institute on Aging (NIA) according to standard protocols (Katwa, 2003; Zhou et al., 2017; Melzer et al., 2020). Rats were euthanized by intraperitoneal injection of heparin sulfate (1,000 U) and sodium pentobarbital (100 mg/kg). The adequacy of anesthesia was confirmed by the lack of pedal withdrawal reflex, corneal reflex, and motor response to pain stimuli by scalpel tip. Fibrotic aged hearts were perfused using the standard Langendorff retrograde perfusion method at 12 ml/min, 37°C with Ca^2+^-free Tyrode's solution for 4 min to remove blood from the vessels, then with enzyme solution for 35–37 min, and finally with 0.05 mmol/l-Ca^2+^ Tyrode's solution to wash out the enzyme solution. The enzyme solution was a Ca-free Tyrode's solution containing 2.2 mg/ml of collagenase (Type II, Worthington) and 0.17 mg/ml of protease (type XIV, Sigma). Digested hearts were removed from the perfusion apparatus and gently agitated to dissociate cells. Digested ventricular tissue was filtered through 70-μm cell strainers and was centrifuged (500 g, 5 min) to remove cardiomyocytes. Single ARVFs thus obtained were suspended in growth medium [Dulbecco's modified Eagle's medium (DMEM) 10% fetal bovine serum (FBS)] for seeding.

### Overexpressing Cx43 in HeLa Cells

Stable gap junction-rich HeLa cells expressing Cx43 and the fluorescent reporter yellow fluorescent protein (YFP) were generated using the Sleeping Beauty transposon system with the transposase plasmid pCMV(CAT)T7-SB100 (pSB100X, Addgene #34879). The gene of interest vector pSBi-RP (Addgene #60513) was prepared by removing the endogenous dTomato and subcloning Cx43YFP into pSBi-RP. Cx43YFP expression within this construct was driven by the constitutively active mammalian promoter EF-1a. HeLa cells were co-transfected with both plasmids at a ratio of 20, Cx43YFPpSBiP:1, pSB100X, using the Bio-T transfection reagent (Bioland Scientific). The transfection complex [1 μg of total DNA and 1.5 μl of Bio-T in 50 μl phosphate-buffered saline (PBS)] was applied to HeLa cell cultures with 70% confluency in 35-mm dishes. Two days post-transfection, HeLa cells expressing Cx43YFP were selected by maintaining the transfected cells in a puromycin (1 μg/ml) selection medium for 1 week. Once the selected HeLa cells expressing Cx43YFP reached confluency, they were split at a ratio of 1:4. Thereafter, T25 flasks were seeded with HeLaCx43 cells, and cells were cultured to confluency without puromycin. HeLaCx43 cell stocks were stored at −140°C and used as needed.

### Patterning Cardiomyocyte–Noncardiomyocyte Interface

**Figure 1:**
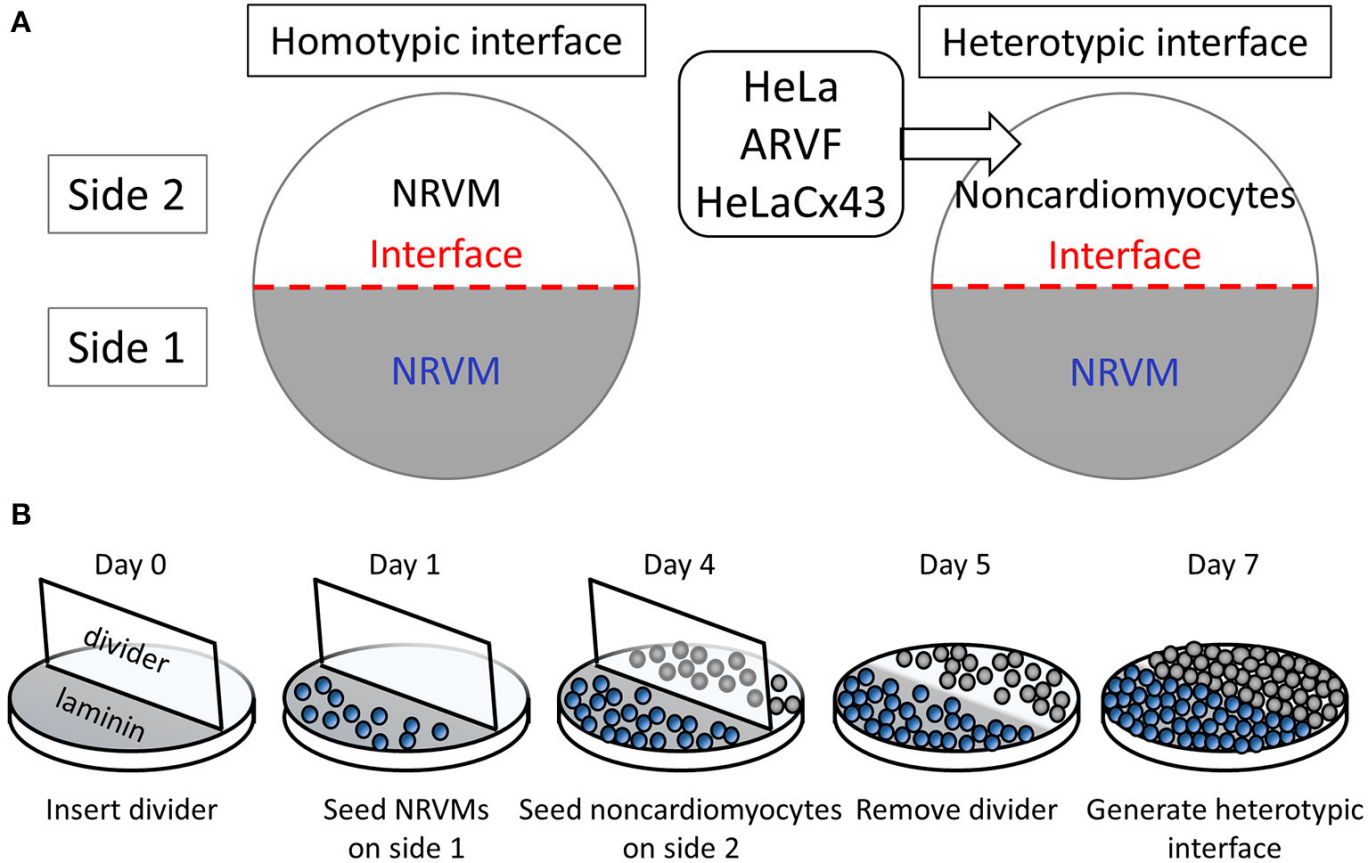
Patterning cardiomyocyte–noncardiomyocyte interfaces. **(A)** Side-1 cell type for all monolayer groups was neonatal rat ventricular cardiomyocytes (NRVMs). Side-2 cell type was either NRVMs in homotypic interfaces (*left panel*) or noncardiomyocytes in heterotypic interfaces (*right panel*). **(B)** The protocol schedule allowed the formation of heterotypic coupling at the interface over 48 h prior to optical mapping of monolayers by day 7.

In our interface design, an interface divides a 14-mm-diameter monolayer into two equal half-circular sides. Side 1 was seeded with Fischer 344 neonatal rat ventricular cardiomyocytes (NRVMs). Side 2 was seeded with either of four cell types with well-known ample or deficient Cx43 expression (Figure 1A). The two side-2 control cell types were Cx43^+^ cardiomyocytes (NRVMs, positive control for robust support of impulse propagation) and Cx43^−^ noncardiomyocytes (HeLa cells, negative control for absent support of impulse propagation). The two side-2 experimental cell types were both Cx43^+^ noncardiomyocytes but from a cardiac vs. non-cardiac source: ARVFs and HeLaCx43 cells.

Monolayers were prepared on 14-mm-diameter glass bottoms inside 35-mm culture dishes (MatTek) following the protocol and schedule depicted in Figure 1B. Because NRVMs grew more slowly than noncardiomyocytes, to pattern our interface model, we used six physical, chemical, and chronological measures to curb successfully the co-cultured noncardiomyocyte overgrowth past the interface into side 1 of NRVMs. (1) During NRVM harvest, neonatal cardiomyocytes were separated from neonatal fibroblasts by centrifugation and plating. (2) Prior to cell seeding, a two-phase aqueous system was created as a chemical barrier by supplementing NRVM medium with dextran (12.8% wt/wt) and noncardiomyocyte medium with polyethylene glycol (5% wt/wt). (3) To promote NRVM adherence and proliferation, glass bottoms were precoated with laminin only on the half-side that would serve as side 1 of heterotypic co-cultures. (4) On day 0, a vertical coverslip was inserted as an additional physical barrier separating the two sides of the dish. (5) On day 1, NRVMs (0.5 million cells/cm^2^) were seeded on the laminin-precoated side 3 days in advance of noncardiomyocyte seeding on day 4 (0.5 million cells/cm^2^). (6) On day 5, the vertical coverslip was removed only in the last 48 h for NRVMs and noncardiomyocytes to make contact at the interface. By day 7, the monolayers were ready for optical mapping.

### High-Speed Optical Imaging

Bright-field microscopy was performed for each monolayer at the start of optical mapping to rapidly locate the heterotypic interface and assess for freedom from macroscopic (large cell clusters as opposed to isolated single cells) invasion of side-1 NRVMs by side-2 noncardiomyocytes. High-speed, high-resolution voltage-sensitive fluorescent optical mapping was performed at 37.0°C after 48 h of co-culture using di-8-ANEPPS (40 μM, 10 min; Invitrogen). Monolayers were superfused at baseline with normal Tyrode's solution and then with H_2_O_2_ solution (100 μM, ≥4 min). The microscope (Nikon) was equipped with a high-speed CMOS camera (100 × 100-pixel resolution; MiCAM Ultima, SciMedia). The 20 × 20-mm^2^ field of view yields a spatial resolution of 200 μm/pixel. Fluorescence was excited with blue light delivered by a 455-nm light-emitting diode through a 550/60-nm excitation filter (Semrock). Spontaneous optical action potentials were recorded at 1.0 ms/frame.

### Data Analysis

We analyzed optical voltage signals using BVAna (SciMedia) as previously described (Nguyen et al., 2016; Zhao et al., 2020). We reduced noise by cubic filtering of signals (3 × 3 pixels). Optical voltage signals were present and analyzed not for HeLa cells but only for the three cell types that supported impulse propagation: NRVMs on either side, and ARVFs or HeLaCx43 cells on side 2.

We measured activation time and activation duration by either one of two quantitative methods: activation map (preferable) or wave display (useful for low peak voltage amplitudes). Wave display is a sensitive method that allows visualization and measurement of multiple selected impulses at once from a single location (selected by one point-cursor), multiple scattered locations (selected by multiple point-cursors), or multiple locations arranged continuously along a selected scanning line. For all 1D wave displays (selected by a point-cursor or a line), time is the independent variable (x-axis). However, the 2D wave display offers the additional information of location as the dependent variable (y-axis), thus a method to calculate the activation duration of the linear cell population along that scanning line. If the peak voltage amplitudes permit, a more elegant and preferable method to calculate activation time and activation duration of a monolayer is to construct an activation map to offer at once a comprehensive bird's-eye view of the entire monolayer (one side or both sides) during only one selected impulse (or depolarization wave). However, because the activation mapping method requires signals of larger amplitudes (such as those generated by NRVMs or HeLaCx43 cells) than the wave display method, not all optical signals (such as those generated by ARVFs) could be mapped. We measured the activation time of a given location in the monolayer at the maximum positive dV/dt of the optical action potential. We measured the activation duration of the entire monolayer (one side or both sides) using activation map for the adequate voltage amplitudes of NRVMs and HeLaCx43 cells and using 2D wave display for the inadequate voltage amplitudes of ARVFs. The activation duration, or the time to activate that entire monolayer, is the difference between the latest and earliest activation time. Because the earliest activation time is the reference time 0 and reflects the focus of activation, the activation duration of a monolayer equals the latest activation time of that monolayer. We calculated NRVM conduction velocity by dividing the monolayer 7-mm radius by NRVM activation duration based on our finding of NRVM transverse conduction anisotropy, from the monolayer edge to the interface (as opposed to longitudinal conduction anisotropy along the monolayer interface).

We measured NRVM action potential duration at 80% of repolarization (APD_80_) as the interval from maximal dV/dt to the moment at 80% of repolarization. Using the APD mapping method, we measured and mapped the APD_80_ distribution during a single excitation wavefront or the mean APD_80_ distribution during multiple excitation wavefronts. Using the wave display method, we calculated the mean of APD_80_ measurements from several successive excitation wavefronts corrected for the spontaneous NRVM beating rate (mean APD_80,c_) by the Bazett formula.

### Statistical Analysis

Statistical analysis was performed using GraphPad Prism8. Continuous variables were expressed as mean ± standard deviation (SD), and categorical variables as percentage. Due to H_2_O_2_ data non-normality as assessed by the Shapiro–Wilk test, non-parametric statistical tests were applied. Statistical significance of differences was evaluated using (1) one-way ANOVA with Tukey's *post-hoc* analysis for multiple-group comparisons of continuous variables under the same condition, (2) the Wilcoxon signed rank test for two-condition comparisons of continuous variables for the same group, and (3) two-tailed Fisher's exact test for comparisons of categorical variables. *P*-value and 95% confidence interval [95% CI] were estimated using the bootstrap-resampling method with 10,000 replications. *P* < 0.05 was the minimal standard for statistical significance.

## Results

Prior to optical mapping, the interface in each heterotypic monolayer was readily located using bright-field microscopy; and macroscopic (large cell clusters as opposed to isolated single cells) invasion of side-1 NRVMs by side-2 noncardiomyocytes was excluded. Single NRVMs are practically indistinguishable from single noncardiomyocytes in a mixture of both cell types. However, in our 2D model, visual distinction of the masses of these two different cell types under bright-field microscopy was readily apparent. Due to inherent cellular density differences, the population of thicker NRVMs appeared more attenuated (whiter) than that of neighboring thinner noncardiomyocytes. Additionally, optical voltage impulses of non-excitable noncardiomyocytes if any were readily distinguished from optical action potentials of excitable NRVMs due to slower upstroke and diminished voltage amplitudes. Thus, besides visual confirmation of the heterotypic interface under bright-field microscopy, this discrepancy in optical voltage signals from excitable NRVMs vs. non-excitable noncardiomyocytes served as additional functional confirmation.

Of note, in this study, we avoided NRVM pacing by design because it can override the noncardiomyocyte-to-myocyte direction of the crosstalk and attenuate or negate potential chaotic electrotonic modulations of NRVMs by noncardiomyocytes and/or H_2_O_2_, such as repolarization impairment, multiple foci of spontaneous activation, or reentry. We found that hyperactivity (multiple rapid foci of spontaneous activation) did not arise in non-paced control NRVMs at baseline. If any, because we took great measures to purify NRVMs from neonatal rat ventricular fibroblasts (NRVFs) following NRVM harvest, non-paced “pure” (NRVF-devoid) NRVM monolayers displayed hypoactivity compared with typical “impure” NRVM monolayers richer in NRVFs.

### Impulse Propagation by Cx43^+^ Noncardiomyocytes Was Passive, Decremental, and Anisotropic

In this section, we focused on side 2 of the monolayer and the forward direction of the crosstalk, from NRVMs to noncardiomyocytes. We aimed to define the impact of NRVMs on noncardiomyocyte electrophysiological behavior, specifically regarding the capability of noncardiomyocytes for impulse propagation. Below, we presented evidence that although Cx43^+^ noncardiomyocytes enabled impulse propagation across the heterotypic interface, impulse propagation by Cx43^+^ noncardiomyocytes was passive, decremental, and anisotropic.

**Figure 2:**
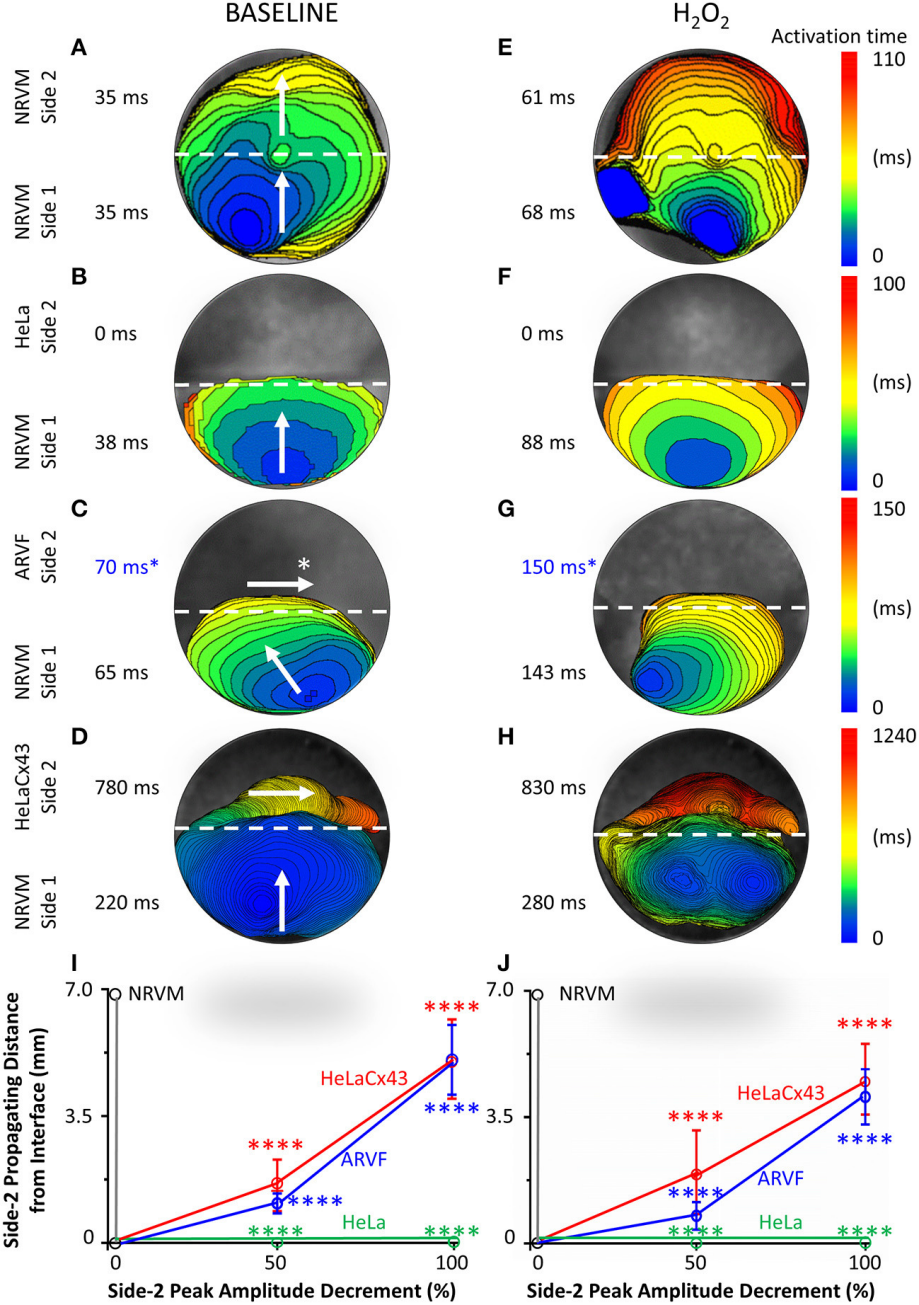
Divergent anisotropies of impulse propagation by cardiomyocytes vs. Cx43^+^ noncardiomyocytes. **(A**–**H)** Representative activation maps (6-ms isochrones) of the entire monolayer under baseline (left column) vs. H_2_O_2_ condition (right column) during a single spontaneous excitation wavefront emerging from side-1 NRVMs, reaching the interface, and with or without success crossing the interface to reach side 2. Note the longitudinal anisotropy of Cx43^+^ noncardiomyocytes (horizontal arrows in **C,D**) in contrast to the transverse anisotropy of NRVMs (vertical/near vertical arrows in **A**–**D**). To ease comparison of the two test conditions for the same monolayer (same row), both activation maps were constructed based on the longer activation duration of the entire monolayer, which was always that under H_2_O_2_ condition. Thus, the four monolayers were mapped based on four different activation-time ranges indicated by numbers next to the color scales. The two activation durations of the two sides in a monolayer were indicated next to the corresponding sides in the monolayer activation map. ^*^ARVF main activation direction and activation durations **(C,G)** were measured using the 2D wave display method (see Supplementary Figure 1). **(I,J)** Under baseline (left panel) vs. H_2_O_2_ condition (right panel), successful propagation of spontaneous action potentials from side-1 NRVMs across the interface to side 2 was supported only by the three Cx43^+^ cell types, but not by Cx43^−^ HeLa cells. Side-2 NRVMs supported active impulse propagation with no amplitude drop-off. In contrast, ARVFs and HeLaCx43 cells supported passive impulse propagation with substantial amplitude decrement. Number of monolayers: NRVM-NRVM, *n* = 9; NRVM-HeLa, *n* = 5; NRVM-ARVF, *n* = 11; NRVM-HeLaCx43, *n* = 8. ^****^P < 0.0001; one-way ANOVA with Tukey's *post-hoc* analysis for multiple-group comparisons under the same condition. *P* > 0.05 for all two-condition comparisons of the same group by the Wilcoxon signed rank test. NRVM, neonatal rat ventricular cardiomyocyte; ARVF, aged rat ventricular myofibroblast.

Figures 2A–H feature four pairs of 6-ms representative isochronal activation maps of four entire monolayers under baseline vs. H_2_O_2_ condition. Each activation map reflects the propagation of a given spontaneous excitation wavefront emerging from side-1 NRVMs, reaching the interface, and with or without success crossing the interface to reach side 2. To ease comparison of the two test conditions for the same monolayer (same row), both maps were constructed based on the longer activation duration of the entire monolayer, which was always under H_2_O_2_ condition. Thus, the four monolayers were mapped on four different ranges of activation times as indicated next to the corresponding color scales. The two activation durations of the two sides in a monolayer were indicated next to the corresponding sides in the monolayer activation map.

Intergroup comparisons of the four monolayers under the same test condition (same column, Figure 2) reveals which of the four side-2 cell types enabled continued propagation of impulses that emerged spontaneously from side-1 NRVMs and crossed the interface. Intercondition comparisons for the same monolayer reveal whether H_2_O_2_ modulated noncardiomyocyte capability to promote impulse propagation.

Under baseline condition, Figure 2A shows that in this NRVM-NRVM monolayer, the entire side-2 NRVM population was activated by an impulse emerging from side 1 in the same duration it had taken to activate the entire side-1 NRVM population. These findings indicate that side-2 NRVMs enabled rapid all-or-none active impulse propagation. In contrast, Figure 2B shows that in this NRVM-HeLa monolayer, the NRVM excitation wavefront from side 1 arrived at the interface but failed to reach the HeLa cells on side 2, indicating that as expected, HeLa cells failed to support impulse propagation. For the NRVM-ARVF monolayer in Figure 2C, ARVF activation by NRVM impulses was evidenced, not by activation mapping but by the more sensitive wave display method (which still did not detect impulse propagation by HeLa cells in Figure 2B). Using 2D wave display (Supplementary Figure 1), we ascertained the main propagation direction by line scan in multiple directions to be from left to right along the interface. The fact that ARVF voltage signals escaped detection by activation mapping underscores that ARVF-mediated impulse propagation was rapidly decremental. Compared with ARVFs in Figure 2C, HeLaCx43 cells in the NRVM-HeLaCx43 monolayer of Figure 2D enabled impulse propagation that decremented less rapidly. However, while impulses propagated through the entire side-2 NRVM monolayer (Figure 2A), impulses propagated through only a portion of the HeLaCx43 monolayer proximal to the interface. Yet it took 255% longer (780 vs. 220 ms) to activate that HeLaCx43 subpopulation than to activate the entire side-1 NRVM population in the same co-culture (Figure 2D). These comparisons underscore that impulse propagation mediated by HeLaCx43 cells was both passive and decremental.

Importantly, compared with side-1 NRVMs in the same monolayer (Figure 2D) or with side-2 NRVMs in an NRVM-NRVM monolayer (Figure 2A), HeLaCx43 cells and NRVMs have divergent anisotropies of impulse propagation, indicating that the electrical connectivity between the two cell types was non-uniform and weak. It is known that anisotropy of impulse propagation has important arrhythmogenic implications as dynamic functional regulation mediated by dynamic gap junctional conductance (Valderrábano, 2007). Within side 1 of Figures 2A–D, following the emergence of an NRVM activation focus in the midline farthest from the interface, the resultant action potentials propagated transversely toward the interface (vertical arrows), indicating that the depolarization wave reached the entire interface length almost simultaneously. In Figure 2A, strong homotypic coupling between NRVMs on both sides of the interface caused simultaneous activation of all side-2 NRVMs along the interface and consequent preservation of the original transverse conduction anisotropy of side-1 NRVMs. In contrast, in Figure 2D, HeLaCx43 cells failed to preserve the transverse conduction anisotropy of side-1 NRVMs. Instead, HeLaCx43 impulses propagated longitudinally along the interface (horizontal arrow) because the HeLaCx43 cells along the interface were not activated by NRVMs simultaneously. Longitudinal stacking of HeLaCx43 activation isochrones indicates that heterotypic NRVM-HeLaCx43 coupling was non-uniform and weaker than homotypic coupling among HeLaCx43 cells. This longitudinal conduction anisotropy was characteristic of all HeLaCx43 co-cultures tested in this study as well as of ARVF co-cultures (as determined by the wave display method; horizontal arrows, Figure 2D and Supplementary Figure 1). Of note, under H_2_O_2_ condition, the divergence in conduction anisotropy between NRVMs and HeLaCx43 cells may not be as apparent because the intrinsic anisotropic conduction properties of both cell types (Valderrábano, 2007) were dynamically perturbed by H_2_O_2_ (Figures 2E–H).

Taken together, at baseline, in the NRVM-NRVM group (*n* = 9), spontaneous action potentials emerging from side 1 propagated actively across the interface to side 2 with preserved peak amplitude; therefore, side-2 NRVMs served as positive control (Figure 2I). This finding indicates that NRVMs on both sides of the homotypic interface were excitable and well coupled to one another. However, in the NRVM-HeLa group (*n* = 5), HeLa cells acted as an electrical barrier to impulse propagation, thus serving as negative control. Subsequent acute H_2_O_2_ exposure did not change the capability of either side-2 control NRVM or HeLa group for impulse propagation (Figure 2J).

In NRVM co-cultures with Cx43^+^ noncardiomyocytes (NRVM-ARVF, *n* = 11 and NRVM-HeLaCx43, *n* = 8), spontaneous NRVM impulses succeeded in propagating across the interface to reach noncardiomyocytes, albeit with decremental peak amplitudes. Peak amplitudes of impulses propagated by ARVFs and HeLaCx43 cells dropped 50% by 1.1 ± 0.3 and 1.6 ± 0.7 mm from the interface and then became extinct by 5.1 ± 1.0 and 5.1 ± 1.1 mm, respectively (Figure 2I). These findings suggest that while Cx43^+^ noncardiomyocytes could act as capacitors for passive NRVM impulse propagation, voltage signals deteriorated, most likely due to non-excitability and weak, variable electrical connectivity to NRVMs. Subsequent acute H_2_O_2_ exposure did not significantly change how far voltage signals could passively propagate past the interface or how fast they deteriorated (Figure 2J). However, in terms of propagating distances and peak amplitudes, HeLaCx43 cells were superior to ARVFs at enabling impulse propagation under both test conditions.

### Cx43^+^ Noncardiomyocytes and H_2_O_2_ Impaired Neonatal Rat Ventricular Cardiomyocyte Activation and Slowed Neonatal Rat Ventricular Cardiomyocyte Conduction

**Figure 3:**
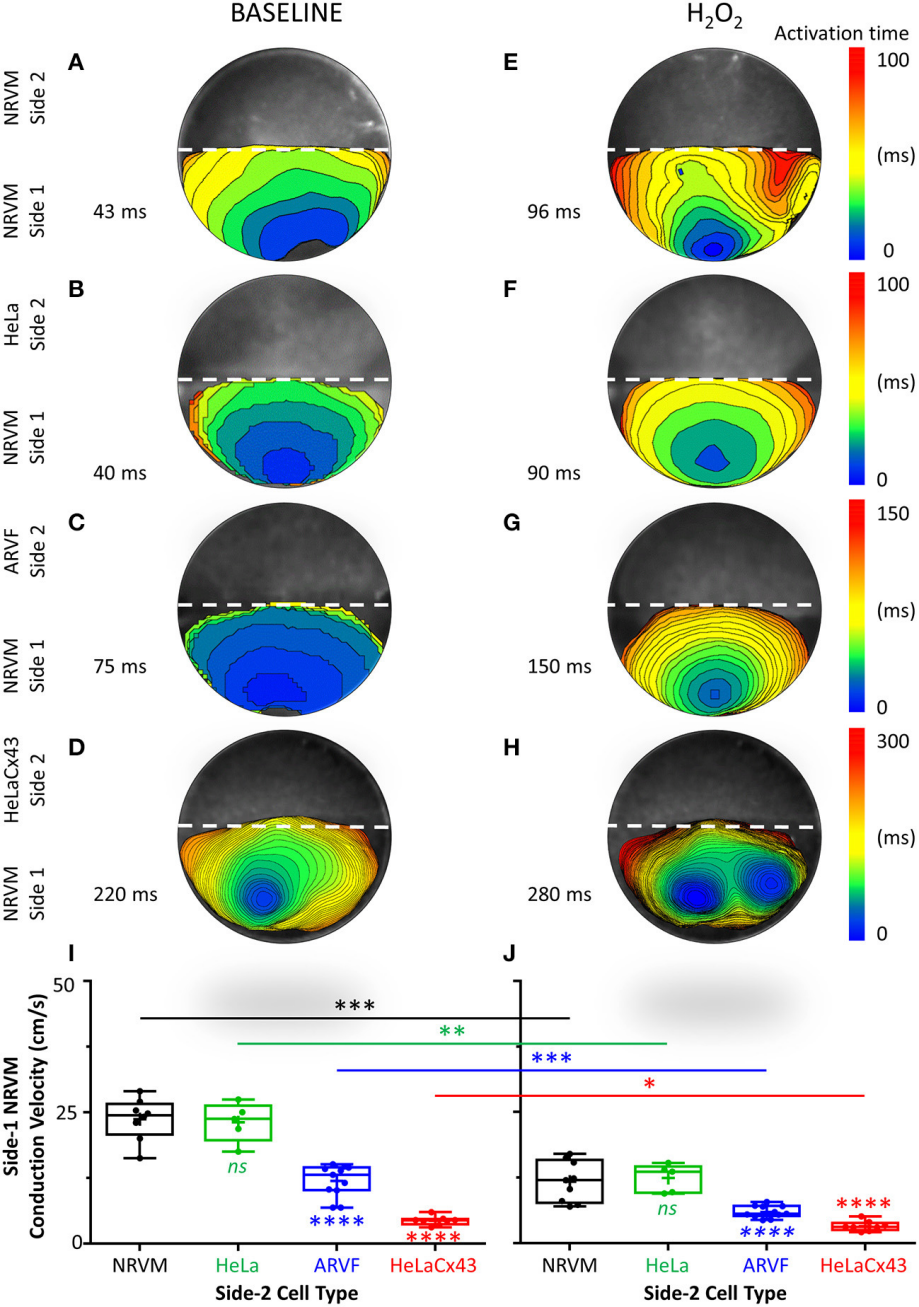
Cx43^+^ noncardiomyocytes and H_2_O_2_ impaired NRVM activation and slowed conduction. **(A**–**H)** Representative activation maps (6-ms isochrones) of side-1 NRVMs under baseline (left column) vs. H_2_O_2_ condition (right column) during a single spontaneous excitation wavefront sweeping across side 1 of the monolayer. To ease comparison of the two test conditions for the same side 1 (same row), both maps were constructed based on the longer of the two side-1 NRVM activation durations, which was always that under H_2_O_2_ condition. Thus, the four monolayers were mapped on four different activation-time ranges indicated by numbers next to the color scales. The activation duration of side 1 in each monolayer was indicated next to its corresponding activation map. Please refer to Supplementary Figure 2 for a summary of independent and synergistic prolongation of NRVM activation duration by Cx43^+^ noncardiomyocytes and H_2_O_2_. **(I,J)** Independent and synergistic slowing of NRVM conduction by Cx43^+^ noncardiomyocytes and H_2_O_2_. Number of monolayers: NRVM-NRVM, *n* = 9; NRVM-HeLa, *n* = 5; NRVM-ARVF, *n* = 11; NRVM-HeLaCx43, *n* = 8. Symbols: box plots with mean (+), median, first and third quartiles (box), minimum and maximum (whiskers). ^*ns*^*P* > 0.05, ^*^P < 0.05, ^**^P < 0.01, ^***^P < 0.001, ^****^P < 0.0001; one-way ANOVA with Tukey's *post-hoc* analysis for multiple-group comparisons under the same condition and the Wilcoxon signed rank test for two-condition comparisons of the same group. NRVM, neonatal rat ventricular cardiomyocyte; ARVF, aged rat ventricular myofibroblast.

In this section, we focused on side 1 of the monolayer and the reverse direction of the crosstalk, from noncardiomyocytes to NRVMs. We aimed to define the impact of Cx43^+^ noncardiomyocytes on NRVM electrophysiological behavior, specifically regarding NRVM activation and conduction. Below, we presented evidence that Cx43^+^ noncardiomyocytes impaired NRVM activation (Figures 2A–H, 3A–H, and Supplementary Figure 2), thereby slowing NRVM conduction (Figures 3I,J), independently and synergistically with H_2_O_2_.

Figures 3A–H features four pairs of representative 6-ms isochronal activation maps of only side-1 NRVMs (not of both sides as in Figures 2A–H) from four monolayers under baseline vs. H_2_O_2_ condition. To ease comparison of the two test conditions for the same side 1 (same row), both maps were constructed based on the longer of the two side-1 NRVM activation durations, which was always that under H_2_O_2_ condition. Thus, the four monolayers were mapped on four different activation-time ranges as indicated next to the corresponding color scales. The activation duration of side 1 in each monolayer was indicated next to its corresponding activation map.

At baseline, neither side-2 NRVMs nor HeLa cells impaired the rapid activation of co-cultured side-1 NRVMs (which measured 35–38 ms in Figures 2A,B and 40–43 ms in Figures 3A,B). In contrast, both ARVFs and HeLaCx43 cells independently prolonged NRVM activation duration by 74–86 and 412–529% over baseline, respectively (to 75 and 65 ms in Figures 2C, 3C and 220 ms in Figures 2D, 3D). These findings indicate that HeLaCx43 cells were more potent than ARVFs at impairing NRVM activation.

In subsequent exposure, H_2_O_2_ independently prolonged the activation duration of side-1 NRVMs in NRVM-NRVM monolayers by 94–123% over baseline (Figure 2E vs. Figure 2A, Figure 3E vs. Figure 3A) and of those in NRVM-HeLa monolayers to similar extent, by 125–132% over baseline (Figure 3F vs. Figure 3B, Figure 2F vs. Figure 2B). Note that the activation durations of NRVMs in co-cultures with HeLa cells (38–40 ms at baseline, 88–90 ms under stress) roughly matched the activation durations of NRVMs on each side of co-cultures with other NRVMs (35–43 ms at baseline; 61–96 ms under stress) under similar test conditions. These findings indicate that HeLa cells exerted no appreciable impact on the co-cultured NRVMs under either test condition. Importantly, H_2_O_2_-induced impairment of NRVM activation was heterogeneous and chaotic as evidenced by the mismatch in isochronal patterns of the same monolayers pre- and post-H_2_O_2_ exposure. Comparisons of Figure 2E with Figure 2A, Figure 3E with Figure 3A, Figure 2F with Figure 2B, and Figure 3F with Figure 3B reveal that H_2_O_2_ did not affect each NRVM in the same isochrone under prior baseline equally.

Evidence that the collective impairment of NRVM activation by both Cx43^+^ noncardiomyocytes and H_2_O_2_ was synergistic came from adding up their individual impairment contributions. For example, independently, ARVFs and H_2_O_2_ prolonged NRVM activation by 86–94% (Figures 2C,E) over baseline (Figure 2A). Therefore, a simple sum of their individual impairment would have amounted to 180%. However, collectively (Figure 2G), they caused instead a synergistic impairment of 309%, or 71% greater than the sum of their individual impairment. Repeating these calculations with the three monolayers in Figures 3A,C,E confirms that, collectively, ARVFs and H_2_O_2_ delayed NRVM activation synergistically by 249%, or 26% greater than expected from the sum of their individual impairment of 198%.

Likewise, the collective impairment of NRVM activation by both HeLaCx43 cells and H_2_O_2_ was also synergistic. HeLaCx43 cells and H_2_O_2_ delayed NRVM activation markedly by 551–700% (Figures 2H, 3H), or 3–12% greater than expected from the sums of their individual impairment of 535% (Figures 3D,E) and 623% (Figures 2D,E) over baseline (Figures 2A, 3A). Note that although H_2_O_2_ synergistic impairment of NRVM activation with HeLaCx43 cells was more severe than that with ARVFs because the contribution by HeLaCx43 cells was larger than by ARVFs, H_2_O_2_ synergy with ARVFs (26 or 71%) was greater than with HeLaCx43 cells (3 or 12%). Importantly, synergistic impairment of NRVM activation by both Cx43^+^ noncardiomyocytes and H_2_O_2_ led to dispersion of NRVM activation, increased NRVM activation gradient, and enhanced NRVM automaticity as evidenced by the emergence of thin, fractionated activation isochrones over longer activation durations, and multiple shifting foci of activation (Figure 3G and Figure 3H vs. Figure 3E and Figure 3F).

Taken together, Cx43^+^ noncardiomyocytes impaired activation of co-cultured NRVMs independently or synergistically with H_2_O_2_ (Supplementary Figure 2), thereby significantly slowing NRVM conduction (Figures 3I,J). However, because HeLaCx43 cells were superior to ARVFs at impairing NRVM activation under both test conditions, HeLaCx43 cells caused greater reduction in NRVM conduction velocity.

### Cx43^+^ Noncardiomyocytes and H_2_O_2_ Impaired Neonatal Rat Ventricular Cardiomyocyte Repolarization and Prolonged Action Potential Duration

Next, we examined the independent and collective impact of Cx43^+^ noncardiomyocytes and H_2_O_2_ on NRVM repolarization and the consequences of such electrical remodeling on NRVM APD_80_.

**Figure 4:**
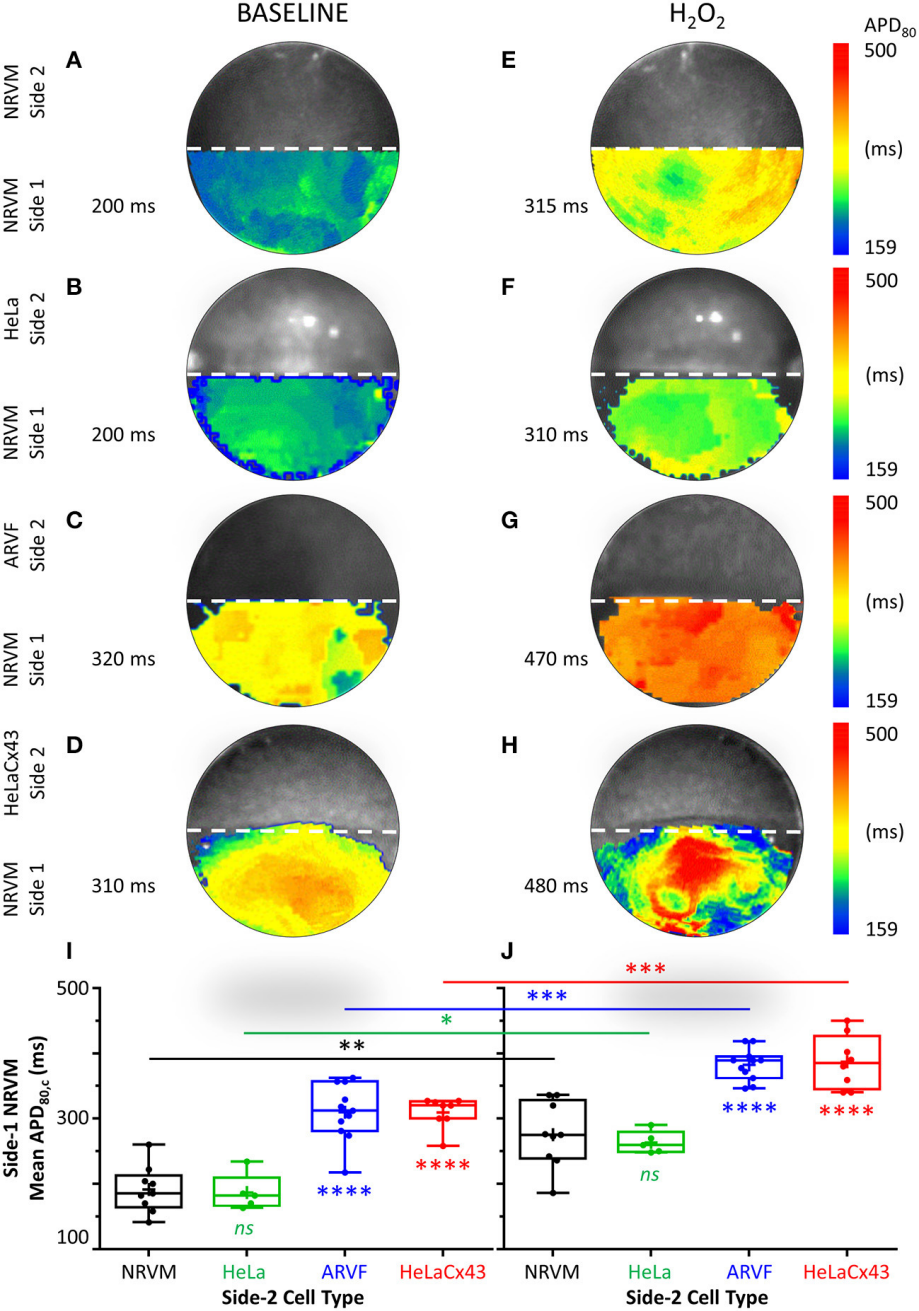
Cx43^+^ noncardiomyocytes and H_2_O_2_ prolonged APD by impairing NRVM repolarization. **(A**–**H)** Representative APD_80_ maps of side-1 NRVMs under baseline (left column) vs. H_2_O_2_ condition (right column) during a single excitation wavefront sweeping across side 1 of the monolayer. To ease intergroup and intercondition comparisons, all maps were constructed based on the longest APD_80_ range of 159–500 ms, which was that of the side-1 NRVM monolayer in co-culture with HeLaCx43 cells under H_2_O_2_ condition. The mean of the APD_80_ distribution was indicated next to the corresponding APD_80_ map. **(I,J)** Means of five APD_80_ measurements from five successive excitation wavefronts corrected for the spontaneous NRVM beating rate by Bazett formula under baseline vs. H_2_O_2_ condition. Note the independent and synergistic prolongation of mean APD_80,c_ by Cx43^+^ noncardiomyocytes and H_2_O_2_. Number of monolayers: NRVM-NRVM, *n* = 9; NRVM-HeLa, *n* = 5; NRVM-ARVF, *n* = 11; NRVM-HeLaCx43, *n* = 8. Symbols: box plots with mean (+), median, first and third quartiles (box), and minimum and maximum (whiskers). ^*ns*^*P* > 0.05, ^*^P < 0.05, ^**^P < 0.01, ^***^P < 0.001, ^****^P < 0.0001; one-way ANOVA with Tukey's *post-hoc* analysis for multiple-group comparisons under the same condition and the Wilcoxon signed rank test for two-condition comparisons of the same group. APD, action potential duration; NRVM, neonatal rat ventricular cardiomyocyte; ARVF, aged rat ventricular myofibroblast.

Figures 4A–H feature four pairs of APD_80_ maps of side-1 NRVMs from four representative monolayers under baseline vs. H_2_O_2_ condition. Each APD_80_ map reflects the APD_80_ distribution within side 1 of a monolayer during a single spontaneous excitation wavefront. Intergroup comparisons of the four monolayers under the same test condition (same column, Figure 4) reveal the modulation impact of noncardiomyocytes. Intercondition comparisons for the same monolayer reveal the modulation impact of H_2_O_2_. To ease intergroup and intercondition comparisons, all maps were constructed based on the longest APD_80_ range of 159–500 ms, which was the same as that of the side-1 NRVM monolayer in co-culture with HeLaCx43 cells under H_2_O_2_ condition. The mean of the APD_80_ distribution was indicated next to the corresponding APD_80_ map.

At baseline, neither side-2 NRVMs nor HeLa cells modulate repolarization of side-1 NRVMs (Figures 4A,B). In contrast, both ARVFs and HeLaCx43 cells impaired NRVM repolarization to similar extent as evidenced by APD_80_ prolongation from control value of 200 ms (Figures 4A,B) to 310–320 ms (a 55–60% prolongation over baseline, Figures 4C,D). Comparing Figures 4E,F with Figures 4A,B reveals that H_2_O_2_ prolonged APD_80_ to 310–315 ms in both control monolayers, indicating that H_2_O_2_ impaired NRVM repolarization and prolonged APD_80_ independently by 58% over baseline. Comparing Figures 4G,H with Figures 4C,D reveals that H_2_O_2_ prolonged APD_80_ synergistically with ARVFs and HeLaCx43 cells at roughly equal extent by 135–140% over baseline, corresponding to 15–24% increase over their respective summative impairment.

Figures 4I,J show the means of APD_80_ measurements from five successive excitation wavefronts corrected for the spontaneous NRVM beating rate by the Bazett formula under baseline vs. H_2_O_2_ condition, respectively. Taken together, impairment of NRVM repolarization by Cx43^+^ noncardiomyocytes independently or synergistically with H_2_O_2_ significantly prolonged APD_80_. However, HeLaCx43 cells and ARVFs were equally capable of impairing NRVM repolarization under both test conditions to cause similar prolongation of NRVM APD_80_.

### Cx43^+^ Noncardiomyocytes and H_2_O_2_ Were Synergistically Arrhythmogenic

Finally, we determined whether independent or synergistic impairment of NRVM activation and repolarization by Cx43^+^ noncardiomyocytes and H_2_O_2_ carries actual arrhythmogenic consequences.

**Figure 5:**
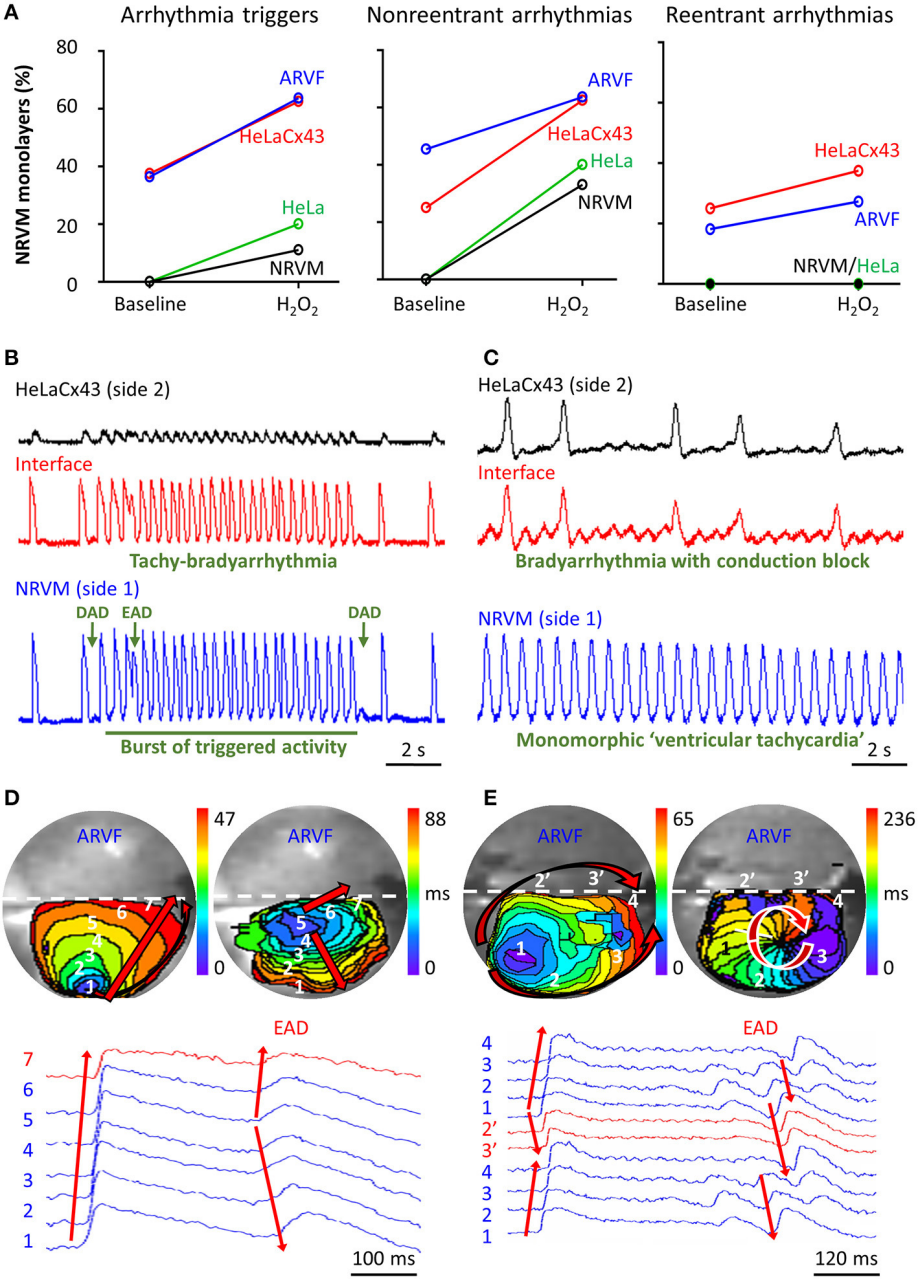
Cx43^+^ noncardiomyocytes and H_2_O_2_ were independently and synergistically arrhythmogenic. **(A)** Cx43^+^ noncardiomyocytes and H_2_O_2_ independently and synergistically increased the likelihood of successful induction of new arrhythmia triggers, and non-reentrant and reentrant arrhythmias. Number of monolayers: NRVM-NRVM, *n* = 9; NRVM-HeLa, *n* = 5; NRVM-ARVF, *n* = 11; NRVM-HeLaCx43, *n* = 8. **(B,C)** Representative optical voltage tracings from two different monolayers illustrate how both HeLaCx43 cells and H_2_O_2_ successfully induced the emergence of new tachy-bradyarrhythmia mediated by arrhythmia triggers **(B)**, monomorphic “ventricular tachycardia,” and bradyarrhythmia mediated by variable conduction block **(C)**. DAD/EAD, delayed/early afterdepolarization **(D,E)** NRVM activation maps (6-ms isochrones) and corresponding 2D wave displays from seven and six different locations within two different NRVM-ARVF monolayers exposed to H_2_O_2_. ARVFs and H_2_O_2_ synergistically induced the emergence of new EAD-mediated non-reentrant arrhythmia (5–4–3–2–1, D right panel) and a reentrant circuit (3–2–1–2′-3′-4, **E** right panel). NRVM, neonatal rat ventricular cardiomyocyte; ARVF, aged rat ventricular myofibroblast.

Figure 5A summarizes the incidence of arrhythmia triggers (left panel) and non-reentrant (middle panel) and reentrant arrhythmias (right panel) of the four groups under baseline vs. H_2_O_2_ condition. The increase of arrhythmia triggers and non-reentrant arrhythmias from baseline to H_2_O_2_ in the two control groups reflects the independent arrhythmogenicity of H_2_O_2_ (Figure 5A, left and middle panels). The higher incidence of arrhythmia triggers, and non-reentrant and reentrant arrhythmias at baseline in the two experimental groups compared with the two control groups reflects the independent arrhythmogenicity of Cx43^+^ noncardiomyocytes (Figure 5A, all three panels). Note that complex reentry mediated by dynamic functional conduction blocks developed only in the presence of Cx43^+^ noncardiomyocytes (Figure 5A, right panel). However, the probabilities of successful arrhythmogenesis were the highest in the presence of both Cx43^+^ noncardiomyocytes and H_2_O_2_. These findings suggest that Cx43^+^ noncardiomyocytes and H_2_O_2_ must have synergized to reduce NRVM repolarization reserve enough for local arrhythmia triggers to overcome the protective local source–sink mismatch and propagate through the rest of the NRVM monolayers.

Figures 5B,C illustrate the synergistic arrhythmogenicity of HeLaCx43 cells and H_2_O_2_. Spontaneous optical action potentials of NRVMs distal to (blue traces) and proximal to (red traces) the interface with HeLaCx43 cells reveal the new emergence of various arrhythmias in the presence of H_2_O_2_. In Figure 5B, exposure of an NRVM-HeLaCx43 monolayer to H_2_O_2_ induced early (EADs) and delayed afterdepolarizations (DADs) and a burst of triggered activity, causing new tachy-bradyarrhythmia to emerge in NRVMs. In Figure 5C, H_2_O_2_ exposure of another NRVM-HeLaCx43 co-culture shortened NRVM spontaneous cycle length by 4.5-fold compared with baseline, precipitating an equivalent of monomorphic “ventricular tachycardia” in NRVMs distal to the interface. However, proximal to the interface, non-uniform functional conduction block and dispersion of refractoriness precipitated bradyarrhythmia.

Figures 5D,E illustrate the synergistic arrhythmogenicity of ARVFs and H_2_O_2_. Activation maps (6-ms isochrones) of NRVMs in co-cultures with ARVFs reveal the new emergence of non-reentrant and reentrant arrhythmias in the presence of H_2_O_2_.

In Figure 5D left panel, following H_2_O_2_ exposure, a spontaneous excitation wavefront emerging in NRVMs most distal to the interface (location 1) took 47 ms to reach NRVMs most proximal to the interface with ARVFs (location 7). Figure 5D right panel illustrates that subsequently as activation of the NRVM monolayer slow further to 88 ms, an EAD emerged near the interface (location 5), leading to non-uniform conduction block and non-reentrant arrhythmia, which reversed the course of impulse propagation away from the interface (from location 5 to 1). In another NRVM-ARVF co-culture exposed to H_2_O_2_ in Figure 5E, both ARVFs and H_2_O_2_ markedly slowed conduction and caused multiple shifting foci of activation, dynamic conduction block, and dispersion of refractoriness, thereby precipitating the dynamic emergence of a functional circuit of reentrant arrhythmia. In Figure 5E left panel, a major spontaneous excitation wavefront emerged left distally to the interface (location 1) and slowly reached the interface (location 4) after 65 ms via two general pathways, one proximal (1–2′-3′-4) and one distal to the interface (1–2–3–4). Note the conduction block caused by an additional simultaneous minor focus of activation (a small purple isochrone) near location 3. In Figure 5E right panel, as activation of the NRVM monolayer slowed further to 236 ms, a new major focus of activation developed at location 3 (near the previously minor activation focus) and set the stage for a reentry circuit (3–2–1–2′-3′-4). The emergence of reentry indicates that the circuit path was longer than the impulse wavelength.

## Discussion

### Dual Protective and Proarrhythmic Potentials of Myofibroblasts

During myocardial aging or wound healing, myofibroblasts secrete collagen and generate fibrosis. It is common knowledge that structural remodeling by fibrosis rescues myocardial mechanical stability but puts the heart at risk for lethal arrhythmias. Less commonly appreciated is how electrical remodeling by fibrosis accounts for its arrhythmogenicity. Here, we provided functional proof-of-concept for the underlying mechanisms. We demonstrated the dual adaptive and maladaptive contributions of myofibroblasts in rescuing impulse propagation yet impairing neighboring cardiomyocyte electrophysiology and rendering them more vulnerable to arrhythmias. Based on our findings, one may view myofibroblasts as “wannabe” cardiomyocytes, loaded with good intentions for structural and electrical remodeling but with imperfect means for successful risk-free execution.

For myofibroblasts in myocardial fibrosis, such as ARVFs, Cx43 was shown to play a critical role in their pathological differentiation from fibroblasts (Asazuma-Nakamura et al., 2009). It was well-established in the literature that myofibroblasts readily form functional gap junctions with cardiomyocytes *in vitro* (Jacquemet and Henriquez, 2008; Pedrotty et al., 2008; McCain et al., 2012; Nagaraju et al., 2019) and *in situ* (Driesen et al., 2005; He et al., 2011; Quinn et al., 2016). Once electrotonically coupled to cardiomyocytes, noncardiomyocytes can serve as sinks for electrotonic currents (Fast et al., 1996). Consistent with that observation, we found that, unlike Cx-deficient HeLa cells, myofibroblasts at the interface with NRVMs enabled 2D impulse conduction that would not have otherwise been possible. Thus, myofibroblasts can potentially rescue cardiac electrical activity by reconnecting electrically separate islands of surviving cardiomyocytes separated by collagen strands and dead cardiomyocytes. On the converse, we found that, unlike excitable NRVMs, non-excitable myofibroblasts at the interface with NRVMs impaired NRVM activation and repolarization. Thus, the cardiac electrical activity rescued by myofibroblasts was decremental, proarrhythmic, and maladaptive.

Our current work provides evidence supporting our hypothesis that arrhythmogenicity is not exclusive to myofibroblasts but pertains also to other Cx43^+^ noncardiomyocyte types. HeLa cells are non-excitable cells that do not express Cx43 (Elfgang et al., 1995; Mesnil et al., 1995). By contrasting HeLa cells with HeLaCx43 cells, we demonstrated that Cx43 overexpression is necessary for non-excitable cells other than myofibroblasts to interface electrically with neighboring excitable cardiomyocytes and act as a capacitive load. This explains why in the normal heart, fibroblasts, which are Cx43-poor, hardly present any arrhythmogenic risk for neighboring cardiomyocytes. Cx43 expression is necessary for noncardiomyocytes not only to form heterotypic gap junction coupling (or nanotube coupling) with cardiomyocytes but also to form homotypic gap junction coupling among themselves for continued propagation of the depolarization wavefront across the noncardiomyocyte population to reach other neighboring viable cardiomyocytes downstream. The functional evidence in this study indicates that the limiting factor for passive electrical conduction was the heterogeneous heterotypic coupling strength along the interface rather than the homotypic coupling strength among HeLaCx43 cells. This observed difference in homotypic vs. heterotypic coupling strength is consistent with prior reports that homomeric–homotypic and homomeric–heterotypic channels differ markedly in their static and dynamic properties of transjunctional conductance and voltage, which dictate their different impulse-propagation capabilities and their respective symmetry vs. asymmetry of electrophysiological functions (Desplantez et al., 2004, 2007; Schulte et al., 2008). Another possible explanation for the difference in homotypic vs. heterotypic coupling strength is that homotypic coupling formation had 1-day advantage over heterotypic coupling formation (3 vs. 2 days, respectively; Figure 1B).

Our group and others (Zlochiver et al., 2008; Vasquez et al., 2010; Nguyen et al., 2012, 2015; Salvarani et al., 2017) previously demonstrated that myofibroblasts can be arrhythmogenic for neighboring cardiomyocytes. We show here that Cx43^+^ non-excitable cells, including myofibroblasts, can impair cardiomyocyte activation and repolarization. This maladaptive role of myofibroblasts contributes to proarrhythmic electrical remodeling by fibrosis. Adult cardiomyocytes have hyperpolarized resting membrane potential ranging from −90 to −80 mV (Pinnell et al., 2007). NRVMs in monolayers have resting membrane potential ranging −80 to −70 mV as recorded by others (Chan et al., 2010) and our group (unpublished data). In contrast, myofibroblasts have depolarized (less negative) resting membrane potentials, widely ranging anywhere from −50 to −5 mV, depending on local microenvironments (Kiseleva et al., 1998; Kamkin et al., 2005). Like myofibroblasts, HeLaCx43 cells have depolarized resting membrane potentials, also widely ranging from −50 to −15 mV (Stein et al., 1996; Ando et al., 2009; Zhang et al., 2009; Hsu et al., 2010). By comparing co-cultures of cardiomyocytes with other cardiomyocytes vs. with Cx43^+^ noncardiomyocytes, we found that a transjunctional cardiomyocyte–noncardiomyocyte gradient of resting membrane potentials is a sufficient condition for noncardiomyocytes to increase cardiomyocyte susceptibility to arrhythmias. This experimental finding validates our earlier prediction in a prior mathematical modeling study (Nguyen et al., 2012).

### Dual Hits by Fibrosis and H_2_O_2_ for Arrhythmogenic Synergy

Here, simulating *in situ* fibrosis in the native heart, we found that *in vitro* interfaces between Cx43^+^ noncardiomyocytes and cardiomyocytes created structural and functional heterogeneities, thereby exerting both passive and active arrhythmogenicity. Heterotypic interfaces lowered the protective source–sink mismatch (Xie et al., 2010; Nguyen et al., 2014) to create a vulnerable substrate (passive arrhythmogenicity). In addition, cardiomyocyte–noncardiomyocyte electrical crosstalk facilitates the initiation and maintenance of arrhythmias by promoting automaticity, causing dynamic functional conduction block, slowing conduction, and prolonging APD (active arrhythmogenicity). We demonstrated that slow conduction by cardiomyocyte–noncardiomyocyte electrical crosstalk promotes the development of reentrant circuits within an area as small as 77 mm^2^. Our finding is compatible with the dimension of the smallest microreentrant circuit of 50 mm^2^ ever reported for the human heart (Spach et al., 1988). Since the impulse wavelength is the product of the conduction velocity and the refractory period, slow conduction reduces the wavelength. For reentry to succeed, the path length must exceed or at least equal the wavelength. Therefore, by reducing the wavelength, slow conduction reduces the minimal path length required for successful reentry (Antzelevitch and Burashnikov, 2011). This explains why a reentrant circuit can develop within such a small border zone area near the cardiomyocyte–noncardiomyocyte interface.

However, experimentally and clinically, not all fibrotic hearts develop arrhythmias until challenged by stress. For arrhythmia triggers to propagate and initiate arrhythmias, a second hit, such as H_2_O_2_-mediated oxidative stress as used in this study, is often required to lower the protective source–sink mismatch and repolarization reserve sufficiently. Xie et al. elegantly demonstrated the underlying cellular mechanisms of H_2_O_2_ arrhythmogenicity in single adult rabbit ventricular cardiomyocytes (Xie et al., 2009). The authors showed that successful induction of early afterdepolarizations by H_2_O_2_ relies on Ca^2+^/calmodulin kinase II activation to impair inactivation of the Na^+^ current (thereby reducing repolarization reserve) and to enhance the L-type Ca^2+^ current (thereby reversing repolarization). Following cardiac injury regardless of cause, oxidative stress contributes critically to pathological remodeling by fibrosis and the ensuing increased proarrhythmic risk at fibrotic border zones. Notably, increased oxidative stress to the heart may occur specifically post-infarction (Ide et al., 2001; Venditti et al., 2001; Carpi et al., 2009; Chi et al., 2017; Margaritis et al., 2017) and in heart failure (Nickel et al., 2015; Bertero and Maack, 2018b; Zhou and Tian, 2018). However, increased oxidative stress to the heart may also occur non-specifically in cardiac inflammation (Zhou et al., 2011), metabolic dysregulation (Nickel et al., 2015; Bertero and Maack, 2018a), and aging (Ander et al., 2007; Lisanti et al., 2011; Martín-Fernández and Gredilla, 2016). Oxidative stress causes the release of reactive oxygen species, such as H_2_O_2_. Importantly, H_2_O_2_ is also a well-recognized pathophysiological paracrine factor produced by activated myofibroblasts in the heart, the lungs (Waghray et al., 2005), and malignant tissues (Lisanti et al., 2011). Previously, we and other groups have tested higher (mM-range) experimental H_2_O_2_ concentrations (Nguyen et al., 2012, 2015; Wang et al., 2014). In this study, we chose the H_2_O_2_ concentration of 100 μM for its pathophysiological relevance (Wang et al., 2014). While low sustained H_2_O_2_ levels of 10–20 μM are routinely produced for baseline cellular signaling (Droge, 2002; Baek et al., 2012), H_2_O_2_ levels during an acute oxidative-stress burst can increase abruptly within 2–6 × 10^−14^ mol/h/cell to hundreds of μM (Granger, 1988; Droge, 2002; Rhee, 2006).

However, we found that the stressor needed as the second hit for arrhythmogenic synergy does not have to be H_2_O_2_ or oxidative stress. We have previously showed that angiotensin II-mediated oxidative stress or hypokalemic stress can also synergize with fibrosis to induce arrhythmias (Bapat et al., 2012; Nguyen et al., 2012; Pezhouman et al., 2015). Other groups have shown that other soluble myofibroblast paracrine factors, such as TGF-β1 (Salvarani et al., 2017) and those from infarcted hearts (Vasquez et al., 2010), can also promote cardiomyocyte–myofibroblast electrical crosstalk and further increased myofibroblast arrhythmogenicity. Our study excluded potential arrhythmogenic contribution by paracrine factors secreted by the cultured myofibroblasts. Instead, we demonstrated that HeLaCx43, which does not secrete myofibroblast paracrine factors at all, can be just as arrhythmogenic as myofibroblasts. Conversely, if HeLa cells secrete different paracrine factors, those factors are not arrhythmogenic by themselves because HeLa cells that do not overexpress Cx43 are not arrhythmogenic.

### Experimental Caveats and Nuances

Several caveats and nuances of the experimental design of this functional study are worth mentioning. Here, we did not seek to reproduce well-established evidence of Cx43 detection at cardiomyocyte–noncardiomyocyte interfaces (Traub et al., 1994; Delorme et al., 1997; Gaudesius et al., 2003; Miragoli et al., 2006; He et al., 2011; Quinn et al., 2016). Instead of seeking indirect speculative structural evidence for heterotypic gap junction/nanotube presence, we provided functional evidence of their existence and, more importantly, their functional consequences.

We chose neonatal, instead of adult cardiomyocytes, as representative cardiomyocytes. While adult cardiomyocytes would offer higher clinical relevance, they unfortunately lose the ability to proliferate in culture to form monolayers. However, we have functional evidence that adult cardiomyocytes retain the ability to form functional heterotypic gap junctions with adult fibroblasts, not only of the same but also of different species, to allow bidirectional passage of macromolecules (Supplementary Figure 3), presumably including ions, which are necessary for bidirectional electrical crosstalk. Therefore, findings using NRVMs in monolayers can be judiciously extrapolated to adult cardiomyocytes in tissue or whole heart settings.

We chose myofibroblasts, instead of fibroblasts, as representative cardiac Cx43^+^ noncardiomyocytes for two reasons: higher clinical relevance and stronger modulation of NRVM electrophysiology. In fact, we initially employed fibroblasts from young adult rat ventricular fibroblasts (YRVFs; 3–6 months of age) in our pilot studies but found negative results, like those with HeLa cells. This finding suggests that at least in our 2D model, while fibroblasts might form functional heterotypic gap junctions with NRVMs (as they did with adult cardiomyocytes shown in Supplementary Figure 3), the net NRVM-fibroblast gap junction current generated did not produce an appreciable arrhythmogenic “source” effect powerful enough to significantly modulate the electrophysiology of the neighboring large NRVM “sink.”

Interestingly, the proarrhythmic electrical remodeling of NRVMs by YRVFs can be captured by a 1D model instead of a 2D model. In our 1D model, 12-mm-long, 1-mm-wide cultured NRVM strands were centrally interrupted by 200-μm-long, 1-mm-wide YRVF inserts (Supplementary Figure 4). YRVFs enabled impulse conduction over a 500-μm distance but markedly impaired NRVM depolarization and repolarization. Consequently, YRVFs slowed NRVM maximal upstroke velocity, delayed NRVM conduction, prolonged APD_75_ and APD_90_, and perturbed impulse propagation. With the synergy from hypokalemic (2 mM) or isoproterenol (1 μM) stress, YRVFs induced early afterdepolarizations and conduction blocks. Our 1D findings are consistent with Gaudesius et al.'s findings in their 1D model of 10-mm-long, 80-μm-wide NRVM strands that centrally located 80-μm-long, 80-μm-wide NRVF inserts permitted impulse conduction over a 300-μm distance (Gaudesius et al., 2003). Interestingly, when HeLaCx43 replaced NRVFs in the inserts, HeLaCx43 cells could double the impulse propagating distance to 600 μm (Gaudesius et al., 2003). Reminiscent of Gaudesius et al.'s finding that HeLaCx43 cells were superior to neonatal fibroblasts in 1D impulse propagating distance, we found that HeLaCx43 cells were superior to ARVFs both in 2D impulse propagating distance (Figure 2) and in NRVM modulation impact, such as more severe NRVM activation delay and greater reduction of NRVM conduction velocity (Figure 3). Nevertheless, what are the underlying determinants? In a prior hybrid biological-computational study of cardiomyocyte–myofibroblast coupling, we identified four critical determinants of higher noncardiomyocyte potential for EAD induction: more depolarized noncardiomyocyte resting membrane potential (which translates to larger transjunctional voltage gradient), larger noncardiomyocyte membrane capacitance and conductance, and larger transjunctional conductance (Nguyen et al., 2012). It is likely that the same four critical factors also played a role to some extent in determining the superior potency of HeLaCx43 cells over ARVFs in certain NRVM proarrhythmic electrical remodeling aspects in our 2D model and over NRVFs in Gaudesius et al.'s 1D model.

Our findings of the contrasting positive vs. negative impacts of YRVFs on NRVM electrical remodeling in the respective 1D vs. 2D model support Xie et al.'s two insightful predictions regarding the importance of structural remodeling on electrophysiological remodeling (Xie et al., 2010). In this classic simulation study, the authors elegantly showed how the minimal numbers of contiguous susceptible cardiomyocytes required to overcome the protective source–sink mismatch for arrhythmia tissue propagation increase drastically as the tissue is scaled up from 1D to 2D and 3D and how all these numbers decrease drastically in the presence of tissue fibrosis. Thus, the presence of heterotypic gap junctions between cardiomyocytes and noncardiomyocytes, even when proven functional, is prognostically meaningless by itself unless properly interpreted in the context of structural remodeling (1D vs. 2D or 3D, absence or presence of fibrosis and local stressors, etc.).

### Clinical Implications for Disease

While our results may have clinical relevance for post-infarction arrhythmogenesis, the clinical relevance of our findings likely extends broadly to arrhythmogenesis in hearts structurally remodeled by fibrosis in general, irrespective of fibrosis etiology. The clinical literature on the mechanisms of arrhythmogenesis at scar border zones in human structural heart disease regardless of cause has demonstrated that conduction of surviving cardiomyocytes at scar border zones is slow and that slow conduction promotes reentrant ventricular tachycardia (De Bakker et al., 1988, 1993; Van Dessel et al., 2001; Patterson et al., 2017). These clinical findings are compatible with two key findings in this study that NRVM conduction at interfaces with noncardiomyocytes is slow and slow conduction promotes reentry.

In a prior review (Nguyen et al., 2012), we discussed that not all myocardial fibrosis patterns are equally arrhythmogenic. Compact fibrosis, characterized by dense areas of collagen and myofibroblasts that are devoid of cardiomyocytes, has the least arrhythmogenicity. In contrast, patchy fibrosis and interstitial fibrosis, associated with border zones of an infarct or a scar in non-ischemic heart disease, have the most arrhythmogenicity. Here, our novel design of cardiomyocyte–noncardiomyocyte interface simulates a short (14-mm) linear scar border zone. The homotypic NRVM monolayer simulates healthy myocardium devoid of structural or electrical remodeling. The heterotypic NRVM-HeLa interface simulates myocardial structural remodeling to demonstrate its effect on passive proarrhythmic electrical remodeling by non-conducting noncardiomyocytes. The heterotypic NRVM-ARVF and NRVM-HeLaCx43 interfaces simulate myocardial structural remodeling to demonstrate its effect on active proarrhythmic electrical remodeling by conducting noncardiomyocytes. The addition of acute H_2_O_2_ exposure simulates how an acute oxidative-stress burst can amplify the combined arrhythmogenicity of structural and electrical remodeling in the fibrotic heart and explain why not all fibrotic hearts develop arrhythmias until challenged by adequate stress.

Our findings further suggest that the strength of gap junction coupling between cardiomyocytes and noncardiomyocytes, if present, is likely to be markedly heterogeneous. Heterogeneous gap junction coupling is an important aspect of tissue inhomogeneity. It was postulated that heterogeneous gap junction coupling contributes critically to ventricular arrhythmogenesis in patients with congestive heart failure (Boulaksil et al., 2011) and non-ischemic dilated cardiomyopathy (Kitamura et al., 2002).

Lastly, while our study concerns ventricular cardiomyocytes, the findings here may also account for the synergistic contribution of myofibroblasts and oxidative stress in the genesis and maintenance of atrial fibrillation in vulnerable fibrotic atrial substrates.

### Clinical Implications for Therapeutics

Our findings may have clinical implications for therapeutics. Like myofibroblasts, exogenous stem cells and progenitor cells (introduced by reparative/regenerative cellular or tissue therapies) express Cx43 and have more depolarized resting membrane potentials than native cardiomyocytes. For example, human induced pluripotent stem cell-derived cardiomyocytes (hiPSC-CMs) have flourished as a powerful tool for drug discovery, disease modeling (Chow et al., 2013; Rocchetti et al., 2017; Chai et al., 2018; Wong et al., 2020), and regenerative medicine (Rhee and Wu, 2018; Sadahiro, 2019; Huang et al., 2020). However, in contrast to adult cardiomyocytes that have stable and more negative resting membrane potential (around −85 mV), hiPSC-CMs do not achieve sufficiently negative membrane potential (−40 to −70 mV), presumably due to inadequate expression of inward rectifier current I_K1_ (Goversen et al., 2018; Horváth et al., 2018). Consequently, although hiPSC-CMs are excitable cardiomyocytes that can provide all-or-none impulse propagation support for endogenous cardiomyocytes, they may have no less arrhythmogenic potential than endogenous myofibroblasts.

Our findings have clinical implications for the engraftment process of cardiac regeneration and tissue engineering therapies. To increase the probability of successful electrical integration, exogenous stem cells and progenitor cells can be engineered prior to transplant to overexpress Cx43. To minimize the arrhythmogenic risk for recipient hearts, exogenous cells can be engineered to overexpress I_K1_ or other stabilizing currents that can hyperpolarize their resting membrane potential to match that of endogenous cardiomyocytes. The caveat is that the three factors—the gradient of resting membrane potentials between noncardiomyocytes and cardiomyocytes, the noncardiomyocyte resistive and capacitive loads, and the cardiomyocyte–noncardiomyocyte electrical coupling strength—all individually and collectively contribute to slowing cardiomyocyte conduction. Therefore, novel antiarrhythmic strategies may need to consider all these three factors to minimize the arrhythmogenic consequences of cell-based or tissue-based cardiac regeneration therapy.

### Study Limitations

This *in vitro* functional proof-of-concept study has limitations. First, although the six precaution measures taken in developing our heterotypic interface model successfully prevented macroscopic invasion of side-1 NRVMs by side-2 noncardiomyocytes, we could not completely exclude microscopic seepage. However, microscopic invasion lacks clinical relevance because isolated single noncardiomyocytes could hardly cause significant impact on the much larger mass of neighboring well-coupled NRVM syncytium. Therefore, the heterotypic interface in our *in vitro* model should not be construed as absolute demarcation dividing cardiomyocytes and noncardiomyocytes. In fact, this design imperfection is superior at recapitulating clinical dynamic 4D cardiac fibrosis border zones, which are not absolute divides between fibrosis and surviving cardiomyocytes. Comparison with the NRVM-HeLa negative control further supports that the low extent of noncardiomyocyte seepage in our interface model made no significant electrophysiological contribution. Second, the interface in our current model was designed to simulate only one specific pattern of structural remodeling, a static 14-mm linear border zone. However, in clinical cardiomyopathies, infinite possibilities of scar border zone patterns exist. By nature, clinical myocardial fibrosis is permanent because it cannot regress in human and other mammalian hearts, which lack the capability to regenerate meaningfully. Yet clinical myocardial fibrosis patterns are transient because they evolve and expand dynamically with time in response to changing local environmental stress and new insults or injuries. This continued evolution of dynamic structural remodeling shapes and is shaped by dynamic electrical remodeling as these two interdependent remodeling processes beget further remodeling. Lastly, this study employed an *in vitro* approach, common to proof-of-concept mechanistic studies. Therefore, additional *ex vivo* 4D studies in intact hearts are necessary to establish relevance to native interfaces of endogenous cardiomyocytes with endogenous noncardiomyocytes (following heart disease, injury, or aging) or with exogenous cardiomyocytes (following cell or tissue transplantation in cardiac regeneration therapies).

## Data Availability Statement

The original contributions presented in the study are included in the article/Supplementary Material, further inquiries can be directed to the corresponding author/s.

## Ethics Statement

The animal study was reviewed and approved by UCLA Institutional Animal Care and Use Committee.

## Author Contributions

SI, MT, and NN performed the experiments. YZ, SI, MT, AL, and NN analyzed and interpreted the data and assisted with manuscript preparation. TN conceived the study, designed the experiments, interpreted the data, and prepared the manuscript. All authors contributed to manuscript revision, approved the submitted version, and agreed to be accountable for all aspects of the work.

## Conflict of Interest

The authors declare that the research was conducted in the absence of any commercial or financial relationships that could be construed as a potential conflict of interest.
