# Supplementary material for: Proarrhythmic Electrical Remodeling by Noncardiomyocytes at Interfaces With Cardiomyocytes Under Oxidative Stress
Source: Front Physiol. 2021 Feb 2;11:622613. doi: 10.3389/fphys.2020.622613 (PMC7884825; doi:10.3389/fphys.2020.622613)
Supplement: Supplementary file 1 [file Image_1.PDF]

Supplementary Material

SUPPLEMENTARY FIGURES

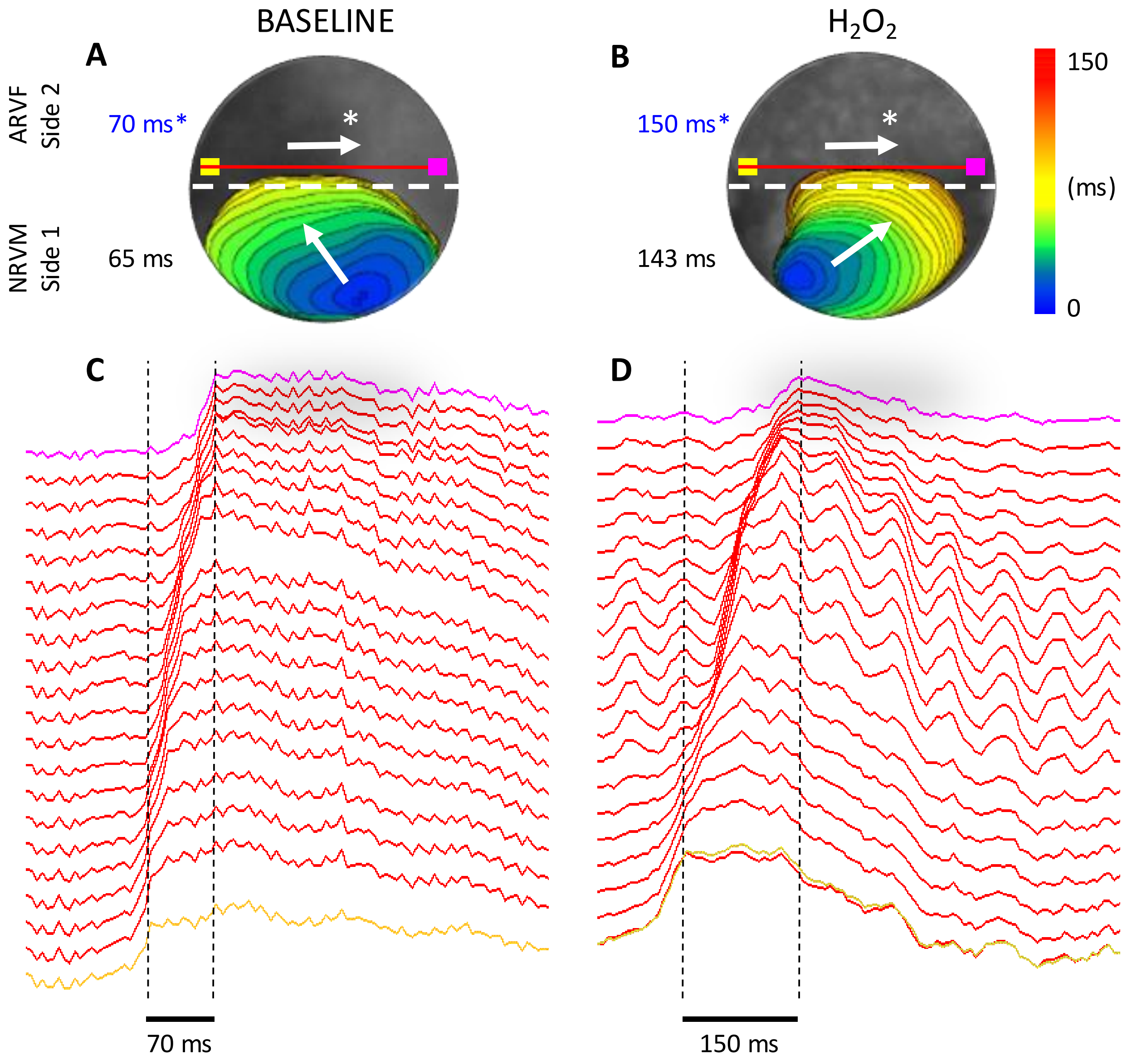

FIGURE S1. Measuring the activation duration of aged rat ventricular myofibroblasts (ARVFs) *by the 2D wave display method. (**A-B**) Reproduction of **Figure 2C, 2G**: In this monolayer of neonatal rat ventricular cardiomyocytes (NRVMs) and aged rat ventricular myofibroblasts (ARVFs), ARVF activation maps could not be constructed to measure ARVF activation durations due to low voltage amplitudes. Instead, ARVF impulse propagation direction and activation duration were determined *by the 2D wave display method. (**C-D**) 2D wave displays of ARVF impulse propagation (x-axis: time; y-axis: location along the line scan). The main propagation direction was ascertained by line scan in multiple directions. The activation duration was calculated from the line scan (red) in the main propagation direction; in this case, along the interface. The yellow and pink cursors distinguish the left and right ends of interface. The yellow and pink waves (corresponding to the left and right cursors) indicate that in this ARVF monolayer, impulses propagated from left to right under both test conditions (ARVF arrows).

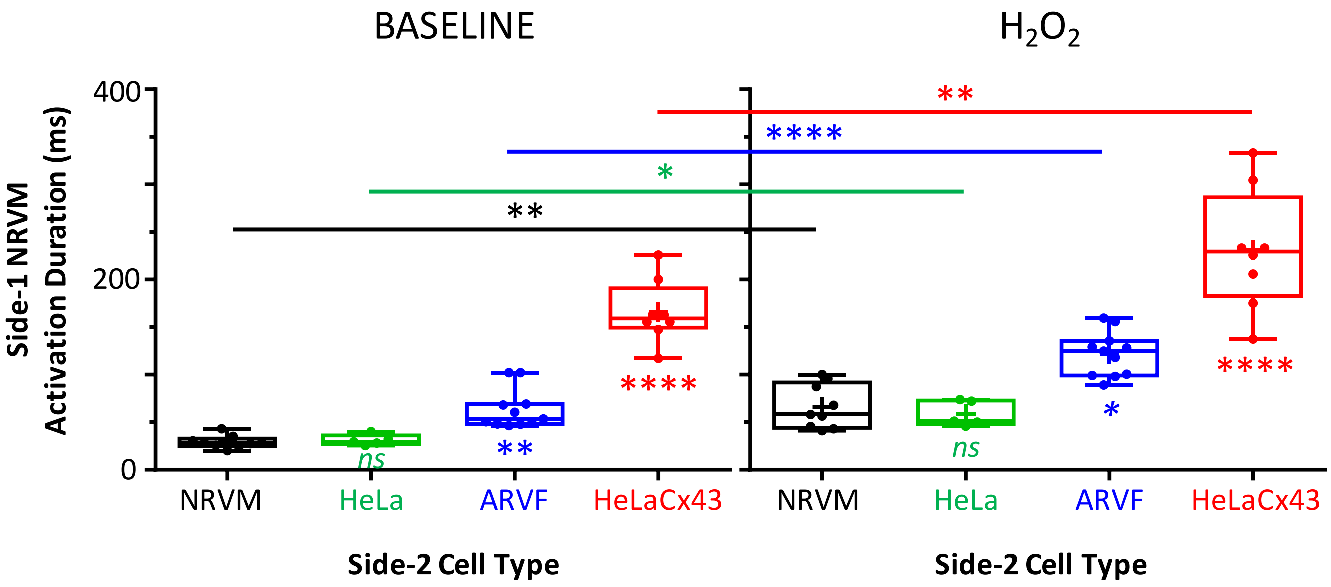

FIGURE S2. Independent and synergistic impairment of NRVM activation by Cx43+ noncardiomyocytes and H_2O_2. Number of monolayers: NRVM-NRVM, *n* = 9; NRVM-HeLa, *n* = 5; NRVM-ARVF, *n* = 11; NRVM-HeLaCx43, *n* = 8. Symbols: box plots with mean (+), median, first and third quartiles (box), minimum and maximum (whiskers). $^{ns}P>0.05$, $^{*}P<0.05$, $^{**}P<0.01$, $^{***}P<0.001$, $^{****}P<0.0001$; one-way ANOVA with Tukey's post-hoc analysis for multiple-group comparisons under the same condition and the Wilcoxon signed rank test for two-condition comparisons of the same group.

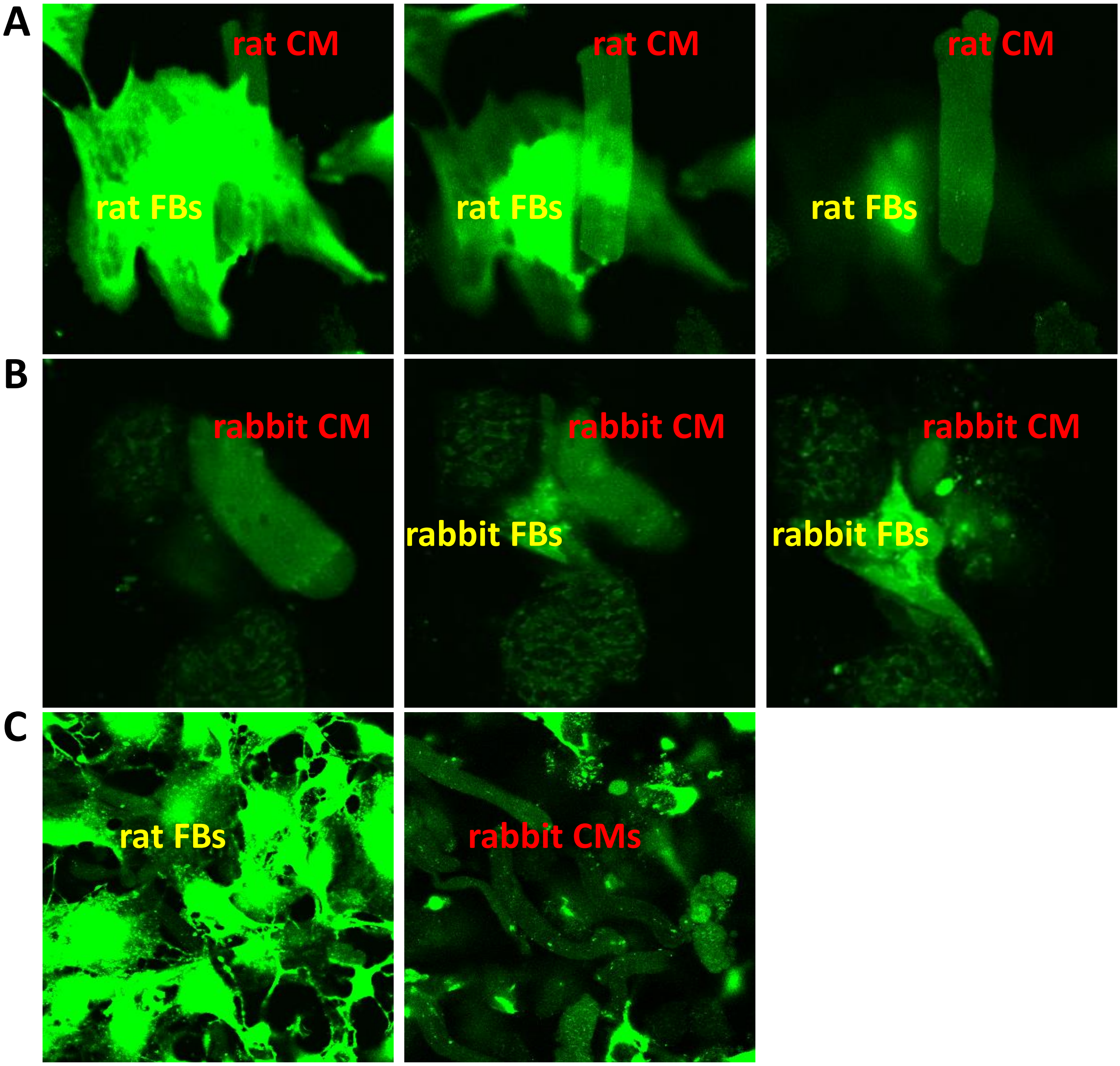

FIGURE S3. Functional gap junction coupling between young adult ventricular cardiomyocytes and young adult ventricular fibroblasts of the same or different species. Confocal laser scanning microscopy (Zeiss LSM5 Pascal) was performed for 24-h cocultures of adult cardiomyocytes (CM) added on top of an adult fibroblast (FB) monolayer to follow the intercellular transport of calcein AM dye (green). (**A**) Calcein AM dye readily transferred from dye-preloaded adult rat FBs to adult rat CMs not dye-preloaded. From left to right: the same coculture section was scanned from bottom to 2-μm, then 6-μm to focus sequentially, first on the FBs, then on both the FBs and coupled CM, and finally on the coupled CM. (**B**) Conversely, calcein AM dye also readily transferred from dye-preloaded adult rabbit CMs to adult rabbit FBs not dye-preloaded. From left to right: the same coculture section was scanned from top to 4-μm, then 7-μm below to focus sequentially, first on the CM then on both the CM and coupled FBs, and finally on the coupled FBs. (**C**) Gap junction coupling can occur not only between different cell types (CM and FB), but also between different species (rat and rabbit). Calcein AM dye readily transferred from dye-preloaded adult rat FBs to adult rabbit CMs not dye-preloaded. From left to right: the coculture was scanned from the bottom to focus first on the rat FBs, then to 5 μm above to focus on the rabbit CMs.

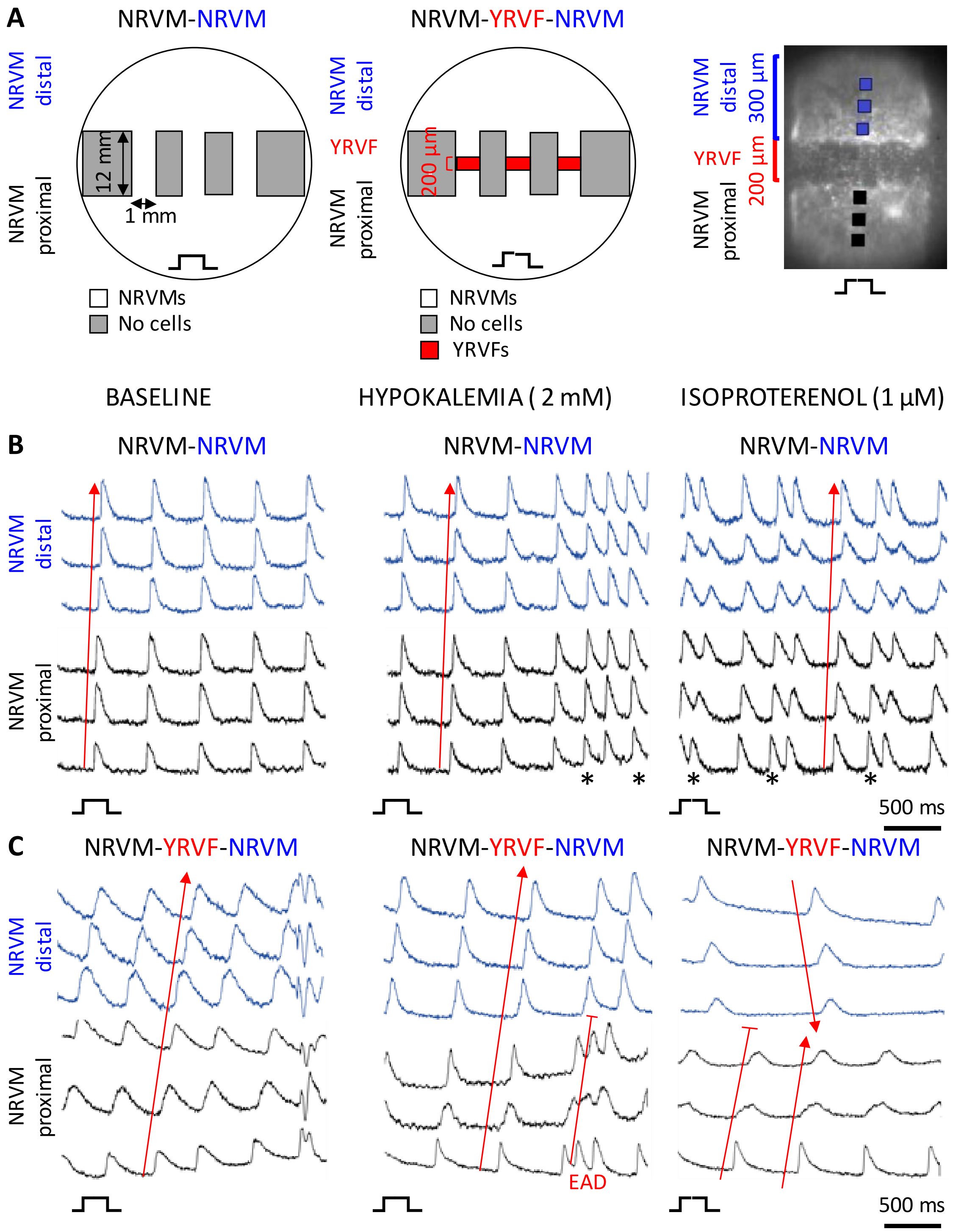

FIGURE S4. Proarrhythmic electrical remodeling of neonatal cardiomyocytes by adult fibroblasts in 1D model. (**A**) Our 1D model consists of 12-mm-long, 1-mm-wide NRVM strands in 35-mm culture dishes. Control NRVM-NRVM strands were uninterrupted. Experimental NRVM-YRVF-NRVM strands were interrupted centrally by a 200-µm-long, 1 mm-wide young rat ventricular fibroblast (YRVF) inserts. YRVFs enabled conduction of 500-ms paced impulses (2-ms, 5-V pacing stimuli) from the proximal NRVMs (black cursors) to propagate 500 µm into the distal NRVMs (blue cursors). Square-pulse-stimulus symbols indicate pacer locations relative to the NRVM strands. Cursors indicate locations of wave sampling. (**B**) In control strands, stress (hypokalemia or isoproterenol) induced new spontaneous triggered activities (*) not otherwise seen at baseline, reflecting the independent NRVM modulation by stress alone. (**C**) In NRVM-YRVF-NRVM strands, YRVFs markedly impaired baseline NRVM activation and repolarization, reflecting the independent NRVM modulation by YRVFs alone. Under stress condition, new early afterdepolarizations (EADs) and conduction blocks emerged, reflecting the synergy between YRVFs and stress.
